# Primary urethral cancer: Treatment patterns, responses and survival in localized, advanced and metastatic patients

**DOI:** 10.1002/bco2.70056

**Published:** 2025-07-17

**Authors:** Ilfad Blazevic, Aude Fléchon, Géraldine Pignot, Benoît Mesnard, Jérôme Rigaud, Mathieu Roumiguié, Michel Soulié, Constance Thibault, Laurence Crouzet, Camille Goislard De Monsabert, Felix Lefort, Marine Gross‐Goupil, Luca Campedel, Mathieu Laramas, Thomas Filleron, Elodie Martin, Léonor Chaltiel, Damien Pouessel

**Affiliations:** ^1^ Oncopole Claudius Regaud Toulouse France; ^2^ Centre Léon Bérard Lyon France; ^3^ Institut Paoli‐Calmettes Marseille France; ^4^ Centre Hospitalier Universitaire de Nantes Nantes France; ^5^ Centre Hospitalier Universitaire de Toulouse Toulouse France; ^6^ Hôpital Georges Pompidou, AP‐HP. Centre‐Université de Paris Paris France; ^7^ Centre Eugène Marquis Rennes France; ^8^ Hôpital Saint‐André, Centre Hospitalier Universitaire de Bordeaux Bordeaux France; ^9^ Groupe Hospitalier Pitié‐Salpêtrière Paris France; ^10^ Centre Hospitalier Universitaire Grenoble France

**Keywords:** adenocarcinoma, chemotherapy, primary urethral cancer, radiation therapy, squamous cell carcinoma, surgery, urothelial carcinoma

## Abstract

**Introduction:**

Primary urethral cancer (PUC) is rare, and limited data exist on optimal treatment and survival, particularly in metastatic cases. The objective of this study was to describe treatment patterns, responses and survival in a contemporary cohort.

**Patients and Methods:**

Data from patients diagnosed with PUC between January 1, 2000 and December 31, 2018, were retrospectively collected from nine French tertiary centres. To enhance the statistical power of survival analysis in the metastatic stage, patients with synchronous and metachronous metastatic disease were pooled.

**Results:**

We identified 71 patients (62% males, 38% females). The most common histological types were urothelial (40.0%), squamous cell (34.3%) and adenocarcinomas (14.3%). At diagnosis, 35.2% had localized disease, 49.3% had locally advanced disease and 15.5% had distant metastases. Twenty‐seven patients had a metachronous metastatic cancer. Multimodal therapy was used in 24% of localized and 57.1% of locally advanced disease. Among the 60 patients with non‐metastatic disease, median disease‐free survival (DFS) was 21.2 months. Nodal involvement was associated with worse DFS (HR: 2.03, p = 0.039), while multimodal treatment did not improve DFS (HR: 1.22, p = 0.5419). For metastatic patients, median overall survival was 15.2 months, and progression‐free survival was 6.4 months. Main study limitations were an overrepresentation of locally advanced disease and the small cohort size.

**Conclusions:**

This retrospective study highlights the significant heterogeneity in terms of histology, stage at diagnosis and treatment of PUC. This study is one of the few to describe treatments and survival in metastatic PUC patients. Efforts must be made to improve survival in these patients.

## 1 INTRODUCTION

Primary urethral cancers (PUC) are rare tumours: with an annual incidence of 1.3 cases per million inhabitants in Europe, they account for less than 1% of all malignancies.^1^, ^2^ The Surveillance of Rare Cancers in Europe (RARECARE) project, which includes only epithelial tumours, shows that incidence increases with age and reveals a male predominance, with a ratio of 3:1.^2^ The most common histological subtype is urothelial carcinoma, followed by squamous cell carcinoma and adenocarcinoma. Identified risk factors include urethral strictures, urethroplasty or urethrostomy,^3^, ^4^, ^5^, ^6^, ^7^ urethral diverticula,^8^, ^9^ HPV infection^10^, ^11^, ^12^ and ionizing radiation.^13^, ^14^ Five‐year relative survival is estimated at 53.8% according to the RARECARE project.^2^ Poor prognostic factors include advanced age,^1^, ^15^, ^16^ black race,^15^, ^16^ the presence of comorbidities,^15^ high T stage or positive N or M stage,^15^, ^16^, ^17^ as well as high‐grade tumours^15^ and histological type.^1^, ^16^, ^18^ Proximal location, N‐positive status and age over 65 years appear to be associated with a higher risk of relapse after treatment.^17^

Managing PUC is challenging due to the heterogeneity of histological subtypes and stages at diagnosis. The European Association of Urology (EAU) and the National Comprehensive Cancer Network (NCCN) guidelines supports a multimodal approach in the locally advanced disease.^19^, ^20^ However, due to the rarity of PUC, no randomized clinical trials have been conducted, resulting in low levels of evidence for these recommendations. Consequently, management is not standardized, and the optimal sequence and combination of treatments remain unclear. Additionally, data on treatment and survival outcomes in metastatic populations are limited.

The objective of this study was to describe, in a contemporary cohort, treatment patterns, response and survival, particularly focusing on the metastatic patients.

## 2 PATIENTS AND METHODS

### 2.1 Study design

Data were retrospectively reviewed and collected from nine French referral centres that are members of the Groupe d'Etude des Tumeurs Uro‐Génitales (GETUG). This study was conducted in accordance with the principles of the Declaration of Helsinki and the General European Data Protection regulation (number 2016/679). The project has been declared to the INDS‐CNIL national directory (number MR2611080120).

### 2.2 Patients

Included patients were males or females over 18 years old diagnosed with PUC between January 2000 and December 2018. Eligible patients must have received at least one treatment in the participating centres. The main exclusion criterion concerned patients with a secondary malignancy of the urethra. Localized disease was defined as Tis‐T2 and N0M0, while advanced disease corresponded to ≥ T3 or N+, M0. The TNM stage used was based on 8th edition of the UICC.^21^

### 2.3 Survival definitions

For non‐metastatic patients, disease‐free survival (DFS) was defined as the time from initial diagnosis to the first occurrence of one of the following events: locoregional or distant recurrence, or death. For any metastatic patients, synchronous or metachronous, progression‐free survival (PFS) was defined as the time from the diagnosis of the metastatic disease to the date of progression or death. Overall survival (OS) was defined as the time from diagnosis to death. Patients without events of interest for each survival definition (recurrence, progression or death) were censored at the date of their last news.

### 2.4 Statistical analysis

Data were summarized by frequency and percentage for qualitative variables and by median and range for quantitative variables. The Kruskal‐Wallis test was used to compare quantitative variables, and the Chi‐square or Fisher's exact test to compare qualitative variables. Survival rates were estimated using the Kaplan–Meier method and presented with their 95% confidence intervals. Univariable survival analyses were performed using the log‐rank test for categorical variables or the Cox proportional hazards model for continuous variables. Hazard ratios (HR) were estimated with 95% confidence intervals. In order to increase the statistical power of survival analysis in the metastatic stage, patients with synchronous and metachronous metastatic disease were pooled. All tests were two‐sided, and p < 0.05 was considered statistically significant. All analyses were conducted with STATA v16 (Stata Corporation, College Station, TX, USA).

## 3 RESULTS

### 3.1 Patients

Seventy‐one patients were identified over the study period. Patients' characteristics are summarized in Table 1. The median age was 65 years. Nearly two‐thirds of the patients (62.0%) were male. Smoking was prevalent in 42.3% of patients. The most common clinical signs included obstructive (42.3%) and irritative (23.9%) symptoms, presence of a mass (32.4%), hematuria (23.9%), extension to the perineum (19.7) and pain (16.9%). Magnetic resonance imaging (MRI) has been used as locoregional assessment in 59.2% patients, while two‐thirds of the patients (66.2%) had a chest computed tomography for distant imaging.

**TABLE 1:** Characteristics of the patients at diagnosis.

	n (%)
**Number of patients**	71 (100)
**Age, years**	
Median	65.0
Range	37.0–87.0
NA	1
**Racial distribution**	
White	57 (89.1)
African	5 (7.8)
Asian	2 (3.1)
NA	7
**Gender**	
Male	44 (62.0)
Female	27 (38.0)
**Medical history**	
Smoking	30 (42.3)
Alcoholism	2 (2.8)
External beam radiotherapy	3 (4.2)
Brachytherapy	0 (0.0)
Urethral stricture	6 (8.5)
Urethritis	0 (0.0)
Cystitis or prostatitis	4 (5.6)
HPV infection	2 (2.8)
Cytotoxic chemotherapy	2 (2.8)
Urethral diverticulum	3 (4.2)
Family history of PUC	1 (1.4)
Exposure aromatic amines	0 (0.0)
Instillation of BCG	2 (2.8)
TURBT	4 (5.6)
TURP	5 (7.0)
**Clinical signs**	
Obstructive symptoms	30 (42.3)
Irritative symptoms	17 (23.9)
Urinary tract infection	6 (8.5)
Hematuria	17 (23.9)
Pain	12 (16.9)
Extension to the perineum	14 (19.7)
Abscess	6 (8.5)
Fistula	5 (7.0)
Mass	23 (32.4)
Lymphadenopathy	3 (4.2)
Incidental	4 (5.6)
**Locoregional imaging**	
MRI	42 (59.2)
CT	48 (67.6)
Ultrasound	16 (22.5)
**Distant imaging**	
TEP	28 (39.4)
Chest CT	47 (66.2)
Bone scintigraphy	19 (26.8)
**Histologic type**	
TCC	28 (39.4)
SCC	24 (33.8)
Adenocarcinoma	10 (14.1)
Melanoma	4 (5.6)
Other or NA	5 (7.0)
**Pathology**	
Grade 1	9 (19.6)
Grade 2	8 (17.4)
Grade 3	29 (63.0)
NA	25
**T stage**	
T0, Tis, Ta, T1	15 (21.1)
T2	16 (22.5)
T3, T4	38 (53.5)
**N stage**	
N0	45 (63.4)
N+	22 (31.0)
**Stage**	
Localized	25 (35.2)
Advanced	35 (49.3)
Metastatic	11 (15.5)
**Site of primary tumour**	
Proximal	40 (58.0)
Distal	29 (42.0)
NA	2
**Site of metastasis (N = 11)**	
Distant lymph node	5 (45.5)
Lung	4 (36.4)
Bone	4 (36.4)
Brain	1 (9.1)
Pleura	1 (9.1)
Liver	0 (0.0)

Abbreviations: SCC = squamous cell carcinoma; TCC = transitional cell carcinoma; PS = performance status; NA = not available; BCG = bacillus Calmette‐Guérin; TURBT = trans urethral removal of bladder tumour; transurethral resection of the prostate; TURP = transurethral resection of the prostate; HPV = human papillomavirus; PUC = primary urethral cancer; MRI = magnetic resonance imaging; CT = computed tomography.

Histological subtypes encountered in this cohort included transitional cell carcinoma (TCC, 39.4%), squamous cell carcinoma (SCC 33.8%) and adenocarcinoma (14.1%). Melanoma was present in 4 patients (5.6%). Significant differences in histological subtypes were observed based on gender (p = 0.0012): adenocarcinoma was more frequent in females (29.6%) compared to males (4.7%), while TCC and SCC were more common in males (48.8% and 41.9%, respectively) compared to females (25.9% and 22.2%, respectively). Among the 46 patients for whom grading data were available, grade 3 was the most frequent, observed in 63.0% of cases. The majority of the patients (49.3%) were diagnosed at an advanced stage, while 35.2% and 15.5% of the patients had a localized and a metastatic disease, respectively. Stages T3 and T4 were seen in 53.5% of patients, and nearly a third (31.0%) had nodal involvement. The tumour was localized to the proximal urethra in 58.0% of patients. Out of the 11 patients with metastatic disease at initial diagnosis, the most common involved sites were distant lymph nodes (45.5%), lung (36.4%) and bones (36.4%).

### 3.2 Treatments in non‐metastatic patients

Definitive treatments for the 60 non‐metastatic patients of the cohort are detailed in Table 2. A multimodal approach was more frequent (57.1%) in advanced stages compared to localized stages (24.0%). Local treatment consisted of radical surgery in the majority of patients (75.0%). A third (33.3%) underwent anterior pelvectomy, and 22.2% had a cystoprostatectourethrectomy. Lymphadenectomy was performed in 75.6% of patients, more frequently in advanced stages (81.5%). Among patients with localized disease, 44.4% were classified as pT3 or pT4. A majority (58.1%) of patients were pN0. Only patients with localized stages underwent a transurethral resection (24.0%). Twenty‐one (35.0%) patients received radiotherapy, most of whom (18 patients) had advanced disease. Radiotherapy was delivered perioperatively in 12 patients (57.1%) and as the exclusive treatment in nine patients (42.9%). Among these nine patients, four (44.4%) received concurrent chemotherapy. Chemotherapy was administered to a third (33.3%) of the patients, mostly in advanced‐stage patients (42.9%), either in neoadjuvant or adjuvant setting.

**TABLE 2:** Definitive treatment in non‐metastatic patients.

	Overall	Localized	Advanced
		T1N0 or T2N0	T3‐T4 or N1
	n (%)	n (%)	n (%)
**Number of patients**	60 (84.5)	25 (35.2)	35 (49.3)
**Modality of treatment**			
Unimodal	34 (56.7)	19 (76.0)	15 (42.9)
Multimodal	26 (43.3)	6 (24.0)	20 (57.1)
**Treatment**			
Transurethral resection	6 (10.0)	6 (24.0)	0 (0.0)
Surgery	45 (75.0)	18 (72.0)	27 (77.1)
Radiotherapy	21 (35.0)	3 (12.0)	18 (51.4)
Chemotherapy	20 (33.3)	5 (20.0)	15 (42.9)
**Chemotherapy details (n = 20)**			
Adjuvant	6 (30.0)	1 (20.0)	5 (33.3)
Neoadjuvant	14 (70.0)	4 (80.0)	10 (66.7)
**Radiotherapy details (n = 21)**			
Radiotherapy as perioperative treatment	12 (57.1)	1 (33.3)	11 (61.1)
Neoadjuvant	2 (17.7)	0 (0.0)	2 (18.2)
Adjuvant	10 (83.3)	1 (100)	9 (81.8)
Radiotherapy as exclusive treatment	9 (42.9)	2 (66.7)	7 (38.9)
Dose, Gy			
Median	60.0	54.5	60.0
Range	35.0–74.0	35.0–74.0	45.0–70.0
Concomitant chemotherapy	4 (44.4)	0 (0.0)	4 (57.1)
**Surgery details (n = 45)**			
Treatment of the tumour			
Urethrectomy (U)	7 (15.6)	4 (22.2)	3 (11.1)
Cystourethrectomy	1 (2.2)	0 (0.0)	1 (3.7)
Cysto (Pr) (U)	10 (22.2)	6 (33.3)	4 (14.8)
Anterior pelvectomy	15 (33.3)	4 (22.2)	11 (40.7)
Penectomy (Pe)	8 (17.8)	3 (16.7)	5 (18.5)
Prostatectomy (Pr)	1 (2.2)	1 (5.6)	0 (0.0)
Cysto (Pr) (U) (Pe)	3 (6.7)	0 (0.0)	3 (11.1)
Lymphadenectomy	34 (75.6)	12 (66.7)	22 (81.5)
**Postoperative pathology (n = 45)**			
Tumour			
pT0, pTis, pT1, pT2	17 (38.6)	10 (55.6)	7 (26.9)
pT3, pT4	27 (61.4)	8 (44.4)	19 (73.1)
Missing	1	0	1
Node			
pNx	9 (20.9)	5 (29.4)	4 (15.4)
pN0	25 (58.1)	11 (64.7)	14 (53.8)
pN+	9 (20.9)	1 (5.9)	8 (30.8)
Missing	2	1	1

### 3.3 Treatments in metastatic patients

The metastatic population consisted of 38 patients, 11 with synchronous and 27 with metachronous metastatic disease. Table 3 summarizes the first three lines of systemic treatments and their respective responses. The number of patients decreased with each subsequent line of treatment: 29 received a first‐line treatment, 17 received second‐line treatment and 11 received a third‐line treatment. The main treatment was chemotherapy, with few patients receiving immunotherapy or targeted therapy. Chemotherapy regimens varied and were guided by the histological subtype, with platinum‐gemcitabine and platinum‐5FU being the most common. The median duration of treatment decreased with the number of lines. The best objective response was progressive disease, with its proportion increasing with each line of treatment: 47.8% for the first‐line, 80.0% for the second‐line and 85.7% for the third‐line of treatment.

**TABLE 3:** Systemic treatments and response in the 38 metastatic patients.

	First‐line	Second‐line	Third‐line
	n (%)	n (%)	n (%)
**Number of patients**	29 (73.3)	17 (44.7)	11 (28.9)
**Type of treatment**			
Chemotherapy	27 (93.1)	13 (76.5)	9 (81.8)
Immunotherapy	2 (6.9)	3 (17.6)	2 (18.2)
Targeted therapy	1 (3.4)	3 (17.6)	1 (9.1)
**Chemotherapy details**			
Regimen			
Platinum gemcitabine	10 (37.0)	2 (15.4)	1 (11.1)
Platinum 5FU	6 (22.2)	1 (7.7)	0 (0.0)
MVAC	2 (7.4)	1 (7.7)	1 (11.1)
Carboplatin paclitaxel	1 (3.7)	0 (0.0)	0 (0.0)
FOLFOX	2 (7.4)	0 (0.0)	1 (11.1)
Paclitaxel	0 (0.0)	2 (15.4)	2 (22.2)
Gemcitabine	0 (0.0)	1 (7.7)	2 (22.2)
Other	6 (22.2)	6 (46.1)	2 (22.2)
Duration of treatment, months			
Median	3.5	1.9	1.2
Range	1.0–6.1	1.0–7.5	0.3–3.7
**Best objective response**			
CR	2 (8.7)	1 (6.7)	0 (0.0)
PR	2 (8.7)	0 (0.0)	0 (0.0)
SD	8 (34.8)	2 (13.3)	1 (14.3)
PD	11 (47.8)	12 (80.0)	6 (85.7)
NA	6	2	4

Abbreviations: NA = not available; CR = complete response; PR = partial response; SD = stable disease; PD = progressive disease.

### 3.4 Survival

**FIGURE 1:**
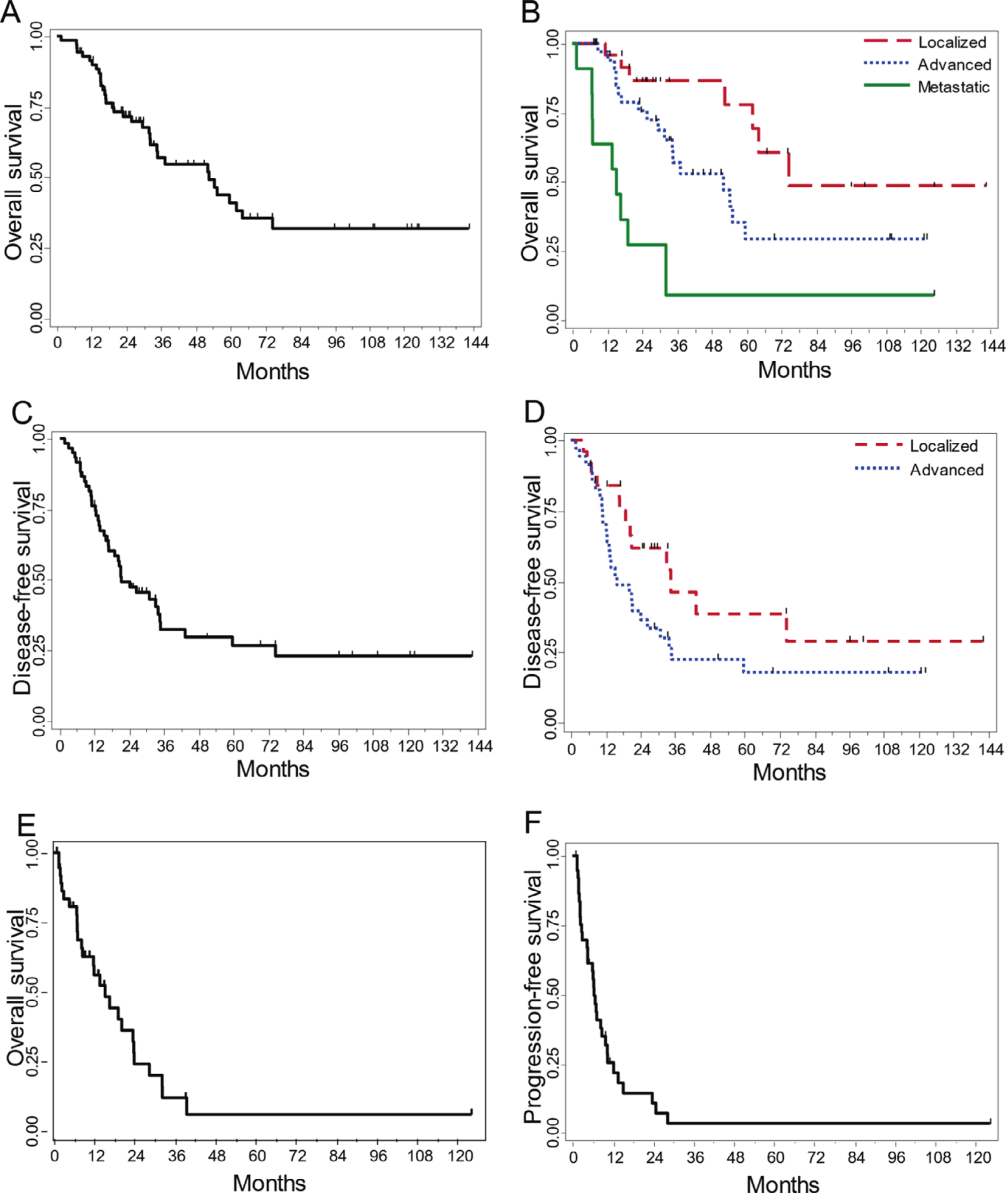
Kaplan–Meier curves for survival. Caption: (A) overall survival in the entire cohort, (B) overall survival according to stage, (C) disease‐free survival in the non‐metastatic patients, (D) disease‐free survival in the non‐metastatic patients according to stage, (E) overall survival in metastatic patients, (F) progression‐free survival in the metastatic patients.

For the entire cohort, the median follow‐up was 66.5 months (95% confidence interval [CI]: 32.8–100.4), and the median OS was 52.6 months (95% CI: 32.2–64.1). Survival rates at one, three and five years were 91.4% [81.8–96.0], 57.1% [43.1–68.8] and 41.1% [26.8–54.8], respectively. The stage at diagnosis was prognostic of death (p < 0.0001). Kaplan–Meier curves for OS are presented in Figure 1, for the entire cohort and according to the stage.

In the 60 non‐metastatic patients, the median OS was 59.6 months (95% CI: 37.3 ‐ not reached [NR]). Survival rates were, at one, three and five years 96.5% [86.7–99.1], 67.3% [51.6–78.9] and 47.5% [30.6–62.7] respectively. The univariable analysis of OS is detailed in Table 4, showing a trend towards worse survival in advanced stages (hazard ratio [HR]: 2.25, 95% CI: 0.93–5.45, p = 0.0647). Site of the tumour, histologic type, grade and treatment modality did not significantly impact OS. Thirty‐nine (65.0%) patients had a progression event, with a median DFS of 21.2 months (95% CI: 16.8–34.5). Kaplan–Meier curves for DFS are presented in Figure 1, and the univariable analysis of DFS is shown in Table 4. Nodal involvement was the only prognostic factor for DFS (HR: 2.03, 95% CI: 1.02–4.05, p = 0.0390). There was a trend for a higher risk of recurrence in the advanced stages (HR: 1.85; 95% CI 0.95–3.61, p = 0.0658). DFS was not impacted by a multimodality treatment (p = 0.5419).

**TABLE 4:** Univariable analysis of survival in non‐metastatic patients.

Variable	Overall survival			Disease‐free survival		
	HR	95% CI	*p*	HR	95% CI	*p*
**Age at diagnosis, years**			0.3792			0.7940
< 65	1.00	‐		1.00	‐	
≥ 65	1.45	0.63–3.31		0.92	0.48–1.75	
**Gender**			0.2393			0.2754
Male	1.00	‐		1.00	‐	
Female	0.61	0.27–1.40		0.70	0.37–1.34	
**T stage**			0.3070			0.7861
T0, T1, T2	1.00	‐		1.00	‐	
T3, T4	1.52	0.68–3.42		1.09	0.58–2.07	
**N stage**			0.1063			**0.0390**
N0	1.00	‐		1.00	‐	
N+	1.95	0.85–4.47		2.03	1.02–4.05	
**Stage**			0.0647			0.0658
Localized	1.00	‐		1.00	‐	
Advanced	2.25	0.93–5.45		1.85	0.95–3.61	
**Site**			0.8353			0.2527
Proximal	1.00	‐		1.00	‐	
Distal	0.92	0.41–2.05		0.69	0.36–1.31	
**Histologic type**			0.9101			0.5134
Adenocarcinoma	1.00	‐		1.00	‐	
SCC	0.87	0.27–2.78		0.79	0.33–1.92	
TCC	0.78	0.25–2.40		0.59	0.24–1.47	
**Pathology**			0.2915			0.2122
Grade 1	1.00	‐		1.00	‐	
Grade 2	1.32	0.31–5.59		1.16	0.37–3.68	
Grade 3	0.53	0.13–2.16		0.51	0.17–1.54	
**Modality of treatment**			0.3785			0.5419
Unimodal	1.00	‐		1.00	‐	
Multimodal	1.44	0.64–3.25		1.22	0.65–2.29	

Abbreviations: HR = hazard ratio; CI = confidence interval; TCC = transitional cell carcinoma; SCC = squamous cell carcinoma;

Regarding the 38 metastatic patients, the median OS was 15.2 months (95% CI: 8.2–23.5) and survival rates were 56.2% [37.9–70.9] and 24.2% [10.2–41.3] at one and two years, respectively. The median PFS was 6.4 months (95% CI: 4.4–9.8 months). Kaplan–Meier curves for OS and PFS are presented in Figure 1. Table 5 shows univariable analyses for OS and PFS in the metastatic population. No factor was significantly associated with OS or PFS. However, a trend towards a higher risk of progression was observed in patients aged 65 years or older (HR; 2.05, 95% CI: 0.97, 4.33, p = 0.0534).

**TABLE 5:** Univariable analysis of survival in the metastatic patients.

Variable	Overall survival			Progression‐free survival		
	HR	95% CI	*p*	HR	95% CI	*p*
**Age at diagnosis, years**			0.4436			0.0534
< 65	1.00	‐		1.00	‐	
≥ 65	1.36	0.62–2.97		2.05	0.97–4.33	
**Gender**			0.3505			0.9404
Male	1.00	‐		1.00	‐	
Female	1.45	0.66–3.19		0.97	0.46–2.04	
**Metastasis occurrence**			0.9634			0.7187
Synchronous	1.00	‐		1.00	‐	
Metachronous	1.02	0.46–2.25		1.15	0.54–2.44	
**T stage**			0.5045			0.9637
T0, T1, T2	1.00	‐		1.00	‐	
T3, T4	1.35	0.56–3.24		1.02	0.48–2.17	
**N stage**			0.6175			0.2605
N0	1.00	‐		1.00	‐	
N+	1.23	0.55–2.76		1.56	0.71–3.40	
**Site**			0.9621			0.7965
Proximal	1.00	‐		1.00	‐	
Distal	1.02	0.47–2.21		1.10	0.53–2.31	
**Histologic type**			0.9741			0.7711
Adenocarcinoma	1.00	‐		1.00	‐	
SCC	0.91	0.27–3.05		0.88	0.30–2.59	
TCC	0.88	0.30–2.61		1.20	0.44–3.26	

Abbreviations: HR = hazard ratio; CI = confidence interval; TCC = transitional cell carcinoma; SCC = squamous cell carcinoma;

## 4 DISCUSSION

This study included all patients with PUC from nine French referral centres over a contemporary period, providing valuable insights into diagnosis, treatment patterns and survival outcomes. Particular focus was placed on metastatic patients, for whom treatment and survival data remain scarce in the literature.

Owing to its rarity, staging in PUC is not standardized and varies across centres. With its high spatial and a contrast resolution, MRI is the most sensitive imaging modality for evaluating local invasion and tumour staging in PUC.^22^ AFU, EAU and NCCN recommend that MRI should be systematically performed for diagnosis.^19^, ^20^, ^23^ In this cohort, only 59% of the patients underwent pelvic MRI. This could be explained by a limited availability of MRI in the early 2000s, and because the first EAU recommendations were issued only in 2013. In this cohort, out of the 18 patients considered initially as localized who underwent radical surgery, 8 patients were finally pT3 or pT4, suggesting potential understating of the disease.

Surgery was the primary treatment for 45 (75%) of the 60 non‐metastatic patients in this cohort. This finding is consistent with other studies, such as an analysis based on 2614 American patients from the National Cancer Database, where surgery was performed on 80% of the patients.^24^ However, in the present cohort, most patients underwent a radical surgery, with only a small number (16%) having urethrectomy. This is probably related to the greater proportion of patients with advanced disease. Transurethral resection concerned only 10% of patients in our cohort, whereas in other series, such as Gakis et al., transurethral resection was performed in 25% of patients.^17^ This discrepancy is also likely attributable to the greater rate of advanced disease in our study.

Regarding recurrences, a high proportion (65%) of the non‐metastatic patients in our cohort experienced a recurrence event. This rate is higher than the 53% observed in Gakis et al.’s cohort, likely due to the higher proportion of advanced stages in our study.^17^ The most frequently involved metastatic sites were the lungs and distant lymph nodes, a finding that has also been reported in another study.^25^

In advanced‐stage disease, the combination of treatments in a multimodal approach aims to reduce recurrences and thus increase survival. Several retrospective studies have shown a benefit on DFS and/or OS from the association of treatments in a multimodal strategy.^24^, ^25^, ^26^, ^27^, ^28^ As a result, both EAU and NCCN guidelines support the use of multimodal treatment in advanced disease.^19^, ^20^ In the present cohort, only 57% of patients with advanced disease patients received multimodal therapy. This study failed to demonstrate a survival benefit from the multimodal treatment, which may be partly due to the high proportion of patients with more advanced disease.

This study is one of the few to describe treatments, response rates and survival outcomes in the metastatic PUC patients. Only a minority of patients achieved an objective response to systemic treatment, and survival remained poor. Comprehensive genomic analysis and screening for clinical trials should be routinely considered for these patients. Jacob and colleagues found that potentially targetable genomic alterations were frequent, involving genes such as *PIK3CA*, *FGFR1*, *FGFR2*, *FGFR3*, *PTEN*, *BRCA1* and *BRCA2*.^29^

This study has several limitations. Due to its retrospective nature, some data were not available. As mentioned above, a selection bias stemming from the inclusion of patients from referral centres resulted in a higher proportion of advanced disease in our cohort. It is possible that the earliest stages (Ta, Tis, T1) are more likely to be treated in general hospitals or clinics, and thus, these patients are underrepresented in our study. Another limitation is the size of the cohort, which has lowered the power of the statistical analyses.

## 5 CONCLUSIONS

This retrospective study provides valuable insights into the diagnosis, treatment patterns and survival of PUC in a contemporary cohort. It is one of the few studies to describe treatment and survival outcomes in metastatic patients. In these patients, low objective response rates and poor prognosis highlight the need for genomic testing and inclusion in clinical trials.

## AUTHOR CONTRIBUTIONS

Ilfad Blazevic had full access to all the data in the study and takes responsibility for the integrity of the data and the accuracy of the data analysis.

Conception and design: Damien Pouessel.

Acquisition of data: Ilfad Blazevic, Aude Fléchon, Géraldine Pignot, Benoît Mesnard, Jérôme Rigaud, Mathieu Roumiguié, Michel Soulié, Constance Thibault, Laurence Crouzet, Camille Goislard De Monsabert, Felix Lefort, Marine Gross‐Goupil, Luca Campedel, Mathieu Laramas.

Analysis and interpretation of data: Ilfad Blazevic, Damien Pouessel.

Drafting of the manuscript: Ilfad Blazevic.

Critical revision of the manuscript for important intellectual content: Aude Fléchon, Géraldine Pignot, Benoît Mesnard, Michel Soulié, Constance Thibault, Felix Lefort, Luca Campedel.

Statistical analysis: Thomas Filleron, Elodie Martin, Léonor Chaltiel.

Obtaining funding: none.

Administrative, technical or material support: none.

Supervision: Damien Pouessel.

## CONFLICT OF INTEREST STATEMENT

All authors declare no conflicts of interest.
